# Identification and characterization of O-GlcNAc modifications of a conserved orthopoxvirus core protein

**DOI:** 10.1128/jvi.00058-25

**Published:** 2025-05-23

**Authors:** Yunliang Zhang, Bernard Moss

**Affiliations:** 1 Laboratory of Viral Diseases, National Institutes of Health, National Institute of Allergy and Infectious Diseases 536965, Bethesda, Maryland, USA; Michigan State University, East Lansing, Michigan, USA

**Keywords:** glycosylation, vaccinia virus, post-translational modification

## Abstract

O-GlcNAcylation, a post-translational modification consisting of O-linked N-acetylglucosamine attached to serine and threonine residues, occurs in thousands of cytoplasmic, nuclear, and mitochondrial proteins but has been reported for relatively few viral proteins. We used click chemistry, specific antibodies, and mass spectrometry to investigate the O-GlcNAcylation of vaccinia virus (VACV) proteins. A virion protein of ~40 kDa was identified by SDS-polyacrylamide gel electrophoresis following azide-alkyne cycloaddition of biotin or an infrared dye to O-GlcNAc residues. Candidate O-GlcNAc virion proteins were detected by mass spectrometry, and A4, a highly conserved core component required for virion assembly, was identified by decreased electrophoretic mobility resulting from the specific attachment of multiple 10 kDa polyethylene glycol residues to O-GlcNAc sites. O-GlcNAc was not detected in virions of an A4 deletion mutant, suggesting A4 is the only or major constituent with this modification. Multiple O-GlcNAc modified amino acids in intrinsically disordered regions of A4 were identified by electron transfer dissociation mass spectrometry. Recombinant A4 was O-GlcNAcylated following stable and transient transfection of uninfected cell lines, suggesting a role for a cellular enzyme, which was confirmed by reduction of the modification by specific inhibitors of O-GlcNAc transferase during virus infection. Moreover, induced degradation of O-GlcNAc transferase prior to VACV infection decreased O-GlcNAcylation of A4 to undetectable levels without diminishing the A4 abundance. Nevertheless, the specific infectivity of O-GlcNAc-deficient virus particles was unimpaired. O-GlcNAcylation either has a subtle role in the VACV life cycle, or A4 is an inadvertent substrate of the promiscuous O-GlcNAc transferase.

**IMPORTANCE:**

O-GlcNAc is a reversible enzymatic post-translational modification of serine and threonine residues found on thousands of cellular proteins with roles in regulating numerous functions including signal transduction, transcription, and stress response. However, little is known about O-GlcNAc modifications of viral proteins. Here, we report that the vaccinia virus A4 core protein has multiple O-GlcNAc modifications. The cellular O-GlcNAc transferase was shown to be required for modifying the vaccinia virus protein, which is synthesized and assembled into virus particles within cytoplasmic virus factories. Moreover, inhibition and degradation of the transferase prevented O-GlcNAcylation of A4. Nevertheless, virus assembly and replication *in vitro* were unaffected by the absence of the modification, suggesting that the addition of O-GlcNAc to A4 has a subtle role or that the modification is a byproduct of a promiscuous O-GlcNAc transferase that preferentially modifies intrinsically disordered regions of proteins.

## INTRODUCTION

Vaccinia virus (VACV), a member of the Orthopoxvirus genus of Chordopoxvirinae, is a large, enveloped virus that replicates entirely in the cytoplasm, has a linear double-stranded DNA genome of approximately 190 kilobase pairs (kbp), and encodes ~200 proteins involved in viral entry, transcription, genome replication, assembly, and host interactions (1). Many orthopoxvirus proteins undergo post-translational modifications including phosphorylation, ubiquitination, and glycosylation (2–4). There are two related forms of poxvirus infectious virions: the intracellular mature virion (MV) and the extracellular virion (EV), which has an additional outer membrane important for exocytosis and virus spread (1). Glycosylation, with one exception, has not been found on proteins comprising the MV but occurs on viral proteins that traffic through the endoplasmic reticulum, particularly those associated with the outer membrane of the EV. The exception is an unidentified ~40 kDa protein component of the MV that was labeled with ^3^H-glucosamine at late times of infection (5, 6). This protein displayed distinct electrophoretic mobility, small oligosaccharide size, and simple carbohydrate composition compared to other virus-induced glycoproteins. Pronase digestion and subsequent acid hydrolysis confirmed glucosamine incorporation. However, unlike typical viral envelope glycoproteins, this particular protein lacked other sugars, namely, fucose, galactose, or mannose, indicating a small carbohydrate modification. In retrospect, we considered that the carbohydrate might be O-linked β-N-acetylglucosamine (O-GlcNAc), which was discovered 13 years after publication of the VACV modification (7).

Over 5,000 eukaryotic proteins with O-GlcNAc modifications have been described in the cytoplasm, nucleus, and mitochondria of eukaryotic cells with suggested roles in the regulation of numerous cellular processes, including signal transduction, transcription, and stress response (8, 9). O-GlcNAcylation is catalyzed by a unique evolutionarily conserved O-GlcNAc transferase (OGT) that transfers a single O-GlcNAc moiety from uridine diphosphate N-acetylglucosamine (UDP-GlcNAc) to serine or threonine residues (10). This modification is reversible, and removal is catalyzed by O-GlcNAcase (OGA) (11). O-GlcNAcylation sites are frequently located in extended loops or intrinsically disordered regions (12), but without a distinctive consensus sequence (13). Despite reports of O-GlcNAcylation of thousands of eukaryotic proteins, O-GlcNAc modifications have been described in relatively few viral proteins (14), namely, the large T antigen of simian virus 40 (SV40) (15), the major tegument protein UL32 of human cytomegalovirus (16, 17), the fiber protein of adenovirus types 2 and 5 (18), the tegument protein of baculovirus (19), the rotavirus nonstructural NS26 protein (20), the coat protein of the potyvirus Plum pox virus (21), and Kaposi’s sarcoma-associated herpesvirus (KSHV) proteins including the transcription factor RTA and proteins of other herpesviruses (22, 23). KSHV RTA is O-GlcNAcylated specifically at Thr366 and Thr367, and mutation of these residues represses its transactivation activity, suggesting a role in the virus life cycle (23). However, O-GlcNAcylation is essential for some cellular processes, making it difficult to distinguish whether diminishing or increasing the OGT activity has a direct or indirect role on virus replication (24).

In the current study, we followed up the >50-year-old report of a VACV 40 kDa protein that was labeled with glucosamine (5). Using advanced click chemistry and mass spectrometry techniques, we determined that the viral A4 core protein required for viral morphogenesis contains multiple O-GlcNAc residues added by the cellular OGT. Induced degradation of OGT prevented O-GlcNAcylation but had no discernible effect on virus infectivity in cultured cells.

## RESULTS

### Detection of a 40-kDa O-GlcNAc virion protein

**Fig 1:**
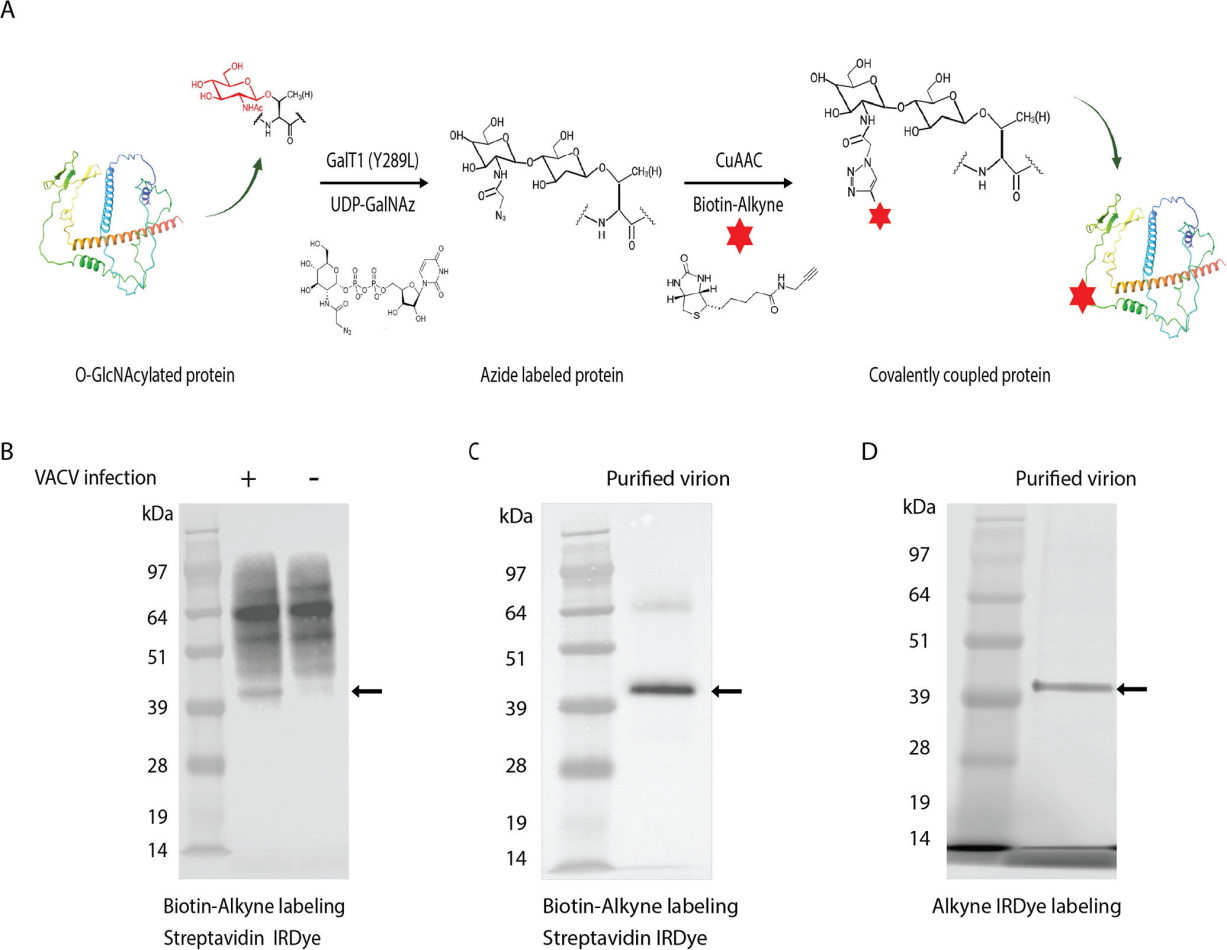
VACV encodes a 40 kDa O-GlcNAc protein. (**A**) Schematic of the click chemistry labeling strategy for O-GlcNAc proteins. O-GlcNAc proteins in cell lysates are tagged with an azide moiety by the mutant β−1,4-galactosyltransferase GalT1 (Y289L) and then with a biotin moiety that has an alkyne functional group by the CuAAC reaction. (**B**) HeLa cells infected with VACV had a detectable 40 kDa O-GlcNAc protein. Lysates of VACV-infected (+) and uninfected (–) HeLa cells were labeled with Biotin-Alkyne by click chemistry, and SDS-PAGE blots were probed with streptavidin IRDye. Marker proteins are shown in the left lane with masses in kDa. The arrow points to the unique O-GlcNAc band in the infected cell lysate. (**C**) Detection of a 40 kDa O-GlcNAc protein in purified VACV virions. Purified virions were solubilized with SDS, and the O-GlcNAc proteins were labeled with Biotin-Alkyne by click chemistry, and SDS-PAGE blots were probed with streptavidin IRDye. The arrow points to a major band. (**D**) Direct visualization of a 40 kDa O-GlcNAc protein in VACV virions using alkyne IRDye. Purified VACV virions were solubilized with SDS, and the O-GlcNAcylated proteins were labeled with the azide moiety followed by click chemistry with alkyne IRDye. The proteins were analyzed by SDS-PAGE, and the wet gel was imaged. The arrow points to a major band.

A chemoenzymatic labeling approach (25) was used to investigate the O-GlcNAcylation of VACV proteins. The method consists of the attachment of N-azidoacetylgalactosamine (GalNAz) to O-GlcNAc residues by an engineered bovine-1,4-galactosyltransferase 1 (Y289L GalT1) (Fig. 1A). The azide group can be linked to various alkyne-modified reagents by copper-catalyzed azide-alkyne cycloaddition (CuAAC), enabling isolation and identification of the modified protein. To implement this approach, proteins in uninfected and VACV-infected HeLa cell lysates were treated with PNGase F to remove GlcNAc at the termini of polysaccharides and reacted with Biotin-Alkyne, as depicted in Fig. 1A. Following SDS-polyacrylamide gel electrophoresis (SDS-PAGE), the blots were probed with IRDye 800CW streptavidin to image biotinylated proteins. In addition to bands detected with proteins of both uninfected and infected cells, there was a biotinylated protein of ~40 kDa specific for the latter (Fig. 1B). Furthermore, a major 40 kDa and a minor >64 kDa protein were detected by reacting SDS-dissociated sucrose gradient purified virions, comprising predominantly of MVs, with Biotin-Alkyne (Fig. 1C). The 40 kDa protein was also detected with an alkyne infrared dye (IRDye) (Fig. 1D). The specificity of the click chemistry reaction strongly suggested that the 40 kDa protein is O-GlcNAcylated and corresponds to the previously identified glucosamine-labeled 40 kDa virion protein (5). The minor higher-molecular weight protein was detected variably and could be an undissociated dimer of the major protein.

### Identification of A4 as the O-GlcNAc protein

**Fig 2:**
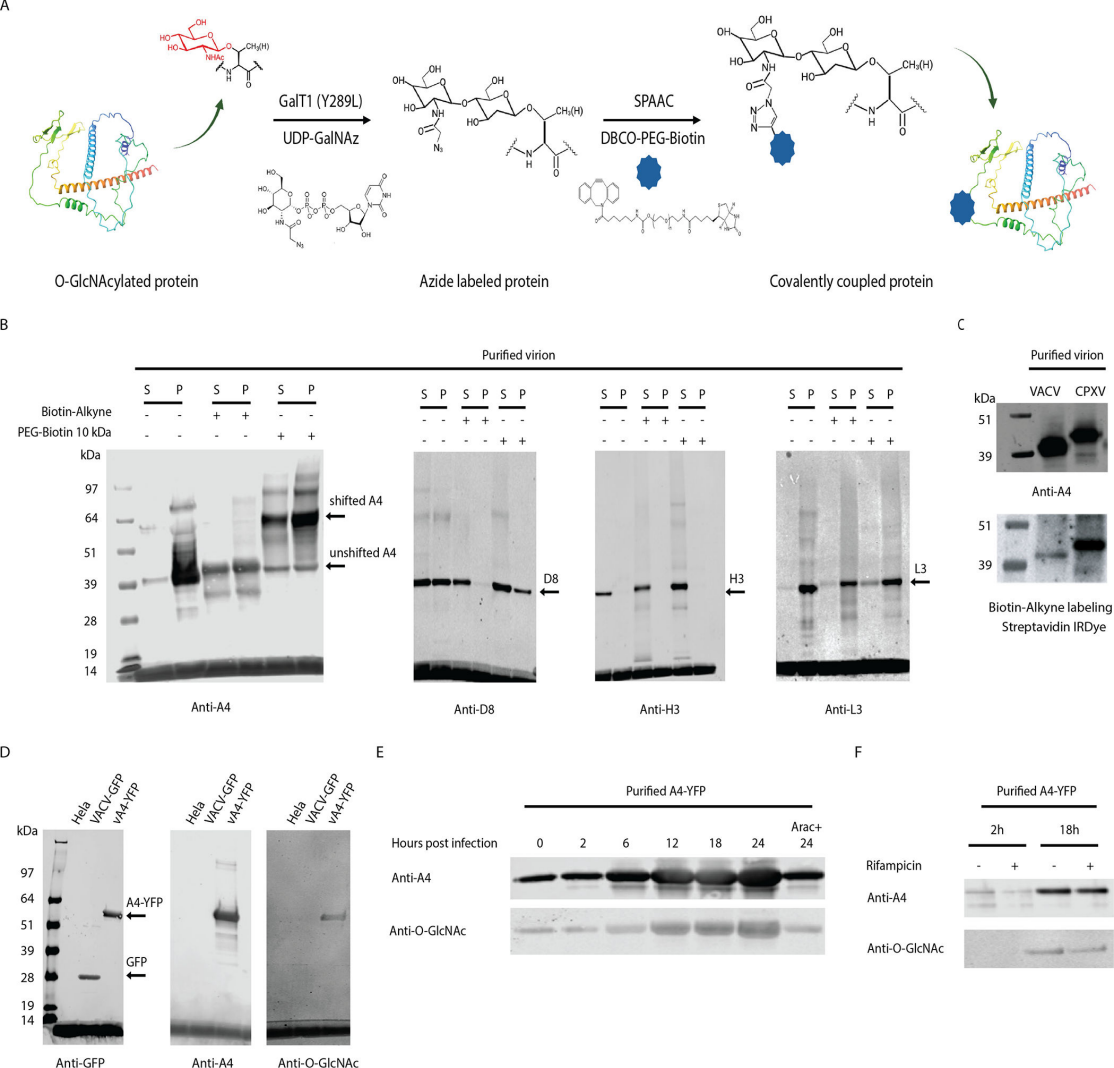
VACV A4 is an O-GlcNAc protein. (**A**) Schematic of the mass tag click chemistry labeling strategy for detection of O-GlcNAc proteins. O-GlcNAc proteins in cell lysates were tagged with the azide moiety by the mutant β−1,4-galactosyltransferase GalT1 (Y289L) followed by the SPAAC reaction with DBCO-PEG-Biotin. (**B**) A4 is the O-GlcNAc component in VACV virions. Purified VACV virions were dissociated with NP-40 buffer, and post-extraction pellets were further treated with SDS. The NP-40 soluble (S) and pellet (P) fractions were labeled with Biotin-Alkyne and PEG-Biotin 10 kDa tag as indicated by (+) and (–). Candidate O-GlcNAcylated proteins were analyzed by SDS-PAGE and immunoblotted using antibodies against the VACV proteins A4, D8, H3, and L3. (**C**) Proteins extracted from purified VACV and CPXV virions were labeled by click chemistry with Biotin-Alkyne and analyzed by SDS-PAGE. Blots were probed with streptavidin IRDye and anti-A4 antibody. (**D**) A4 O-GlcNAc was confirmed by analysis of the purified A4-YFP protein. HeLa cells were infected with VACV-GFP or vA4-YFP, and proteins in the lysates were purified with GFP-Trap magnetic agarose beads. The purified GFP and A4-YFP and proteins of uninfected HeLa cells were analyzed by SDS-PAGE and immunoblotted with antibodies to GFP, A4, and O-GlcNAc. (**E**) O-GlcNAcylation and A4 synthesis occur concomitantly. HeLa cells were infected with vA4-YFP, and at the indicated hours, A4-YFP was purified from cell lysates with GFP-Trap magnetic beads. As a control, A4-YFP was also purified from a parallel culture infected with vA4-YFP for 24 h in the presence of AraC. The purified proteins were analyzed by SDS-PAGE, and immunoblots were probed with antibodies against A4 and O-GlcNAc. (**F**) O-GlcNAcylation of A4 occurs in the presence of the virion assembly inhibitor rifampicin. Cells were infected with A4-YFP in the absence (–) or presence (+) of the drug rifampicin, and purified A4-YFP was analyzed by immunoblotting with specific antibodies.

The putative O-GlcNAc protein was enriched by reacting SDS-dissociated virion proteins with Biotin-Alkyne and capturing the biotinylated product with streptavidin beads. Following elution and trypsin digestion, the peptides were analyzed by liquid chromatography with tandem mass spectrometry (LC-MS-MS). However, the large number of peptides, apparently derived from nonspecific binding of proteins to the beads, impeded the identification of the O-GlcNAc protein by this method. As a second approach, the 40 kDa band linked to the alkyne IRDye (Fig. 1D) was excised from the gel, trypsin-digested, and subjected to MS. Of numerous VACV proteins detected by MS, the four most abundant MV proteins were the cell attachment membrane proteins D8 and H3 and the core proteins A4 and L3 (Table 1). To determine which, if any, of these proteins was O-GlcNAcylated, we employed a mass tag labeling strategy (26). In an adaptation of the method, solubilized virion proteins were chemoenzymatically modified by strain-promoted azide-alkyne cycloaddition (SPAAC). Dibenzocyclooctyne (DBCO)-PEG-Biotin 10 kDa (PEG-Biotin 10 kDa) was attached to azide groups, which in theory should increase the protein mass by 10 kDa for each O-GlcNAc residue (Fig. 2A). Purified virions were treated with the non-ionic detergent NP-40 to extract membrane proteins, and the insoluble pellet fraction was solubilized with SDS. The proteins in each fraction were labeled with Biotin-Alkyne, which has a mass of 281 Da, or with the larger PEG-Biotin 10 kDa moiety and resolved by SDS-PAGE. The proteins were transferred to a membrane, and the blots were probed with antibodies to VACV D8, H3, A4, and L3. Neither the D8, H3, nor L3 proteins exhibited a mass increase with PEG-Biotin 10 kDa (Fig. 2B). However, PEG-Biotin 10 kDa shifted most of the A4 protein from an apparent mass of 40 kDa to 60 kDa and to less abundant bands of ~90 and >100 kDa (Fig. 2B), suggesting several incompletely occupied O-GlcNAc sites. Although the majority of A4 was in the pellet following NP-40 treatment, the proteins of both fractions were labeled with PEG-Biotin. We concluded that A4 has multiple O-GlcNAc residues.

**TABLE 1:** Most abundant virion proteins in the alkyne IRdye band identified by MS

Protein	kDa	Relative abundance	Location and function
D8	35.4	1.08E + 08	Envelope and attachment
H3	37.4	8.23E + 07	Envelope and attachment
A4	30.9	1.24E + 07	Core and structure
L3	40.6	3.22E + 06	Core and transcription

A4 is a highly conserved major core structural protein of orthopoxviruses required for morphogenesis. To determine whether the O-GlcNAc modification occurs within other species of this genus, solubilized proteins of purified cowpox virus (CPXV) virions were subjected to click chemistry labeling. A prominent CPXV 42 kDa band migrating slightly slower than VACV A4 was detected by click chemistry labeling with Biotin-Alkyne and probing immunoblots with anti-A4 antibody (Fig. 2C). The CPXV homolog of VACV A4 has an additional 14 amino acid residues at the N-terminus, accounting for the 2 kDa increase in size compared to that of the VACV A4 protein.

### A4 O-GlcNAc modification occurs concurrently with A4 synthesis and independently of the virion assembly

Previous studies showed that the fusion of green fluorescent protein (GFP) or the closely related yellow fluorescent protein (YFP) to A4 does not perturb its function (27, 28). Additional evidence that A4 is the O-GlcNAc protein was achieved by purification of the A4-YFP fusion protein from HeLa cells infected with a recombinant VACV (vA4-YFP) with GFP-Trap magnetic agarose, followed by immunoblotting with anti-GFP antibody, anti-A4 antibody, and anti-O-GlcNAc monoclonal antibodies (mAbs) (29). As a negative control, cells were also infected with a recombinant VACV expressing GFP (VACV-GFP), which differs from YFP at four amino acids. A protein band of the size expected for the A4-YFP fusion protein was detected with both the anti-A4 and anti-GFP antibodies (Fig. 2D). Taken together, the conjugation of A4 with Biotin-Alkyne, alkyne IRDye, and PEG-Biotin 10 kDa and the binding of A4-YFP fusion protein with anti-O-GlcNAc mAbs provided strong evidence of the O-GlcNAc modification. Detection of HexNAc modifications of A4 obtained by electron transfer dissociation (ETD) mass spectrometry (MS) will be provided in a subsequent section.

A4 is a major structural protein expressed during the late stage of VACV replication and packaged into assembling virions (30). We wanted to determine whether the O-GlcNAc modification is dependent on virion assembly. A time-course experiment with vA4-YFP-infected HeLa cells was carried out. A4-YFP was purified from cell lysates at various times after infection, and SDS-PAGE blots were probed with anti-A4 antibody and anti-O-GlcNAc mAbs. A4-YFP was detected at the 0 time point as the protein is packaged in virus particles and increased in abundance from 6 to 24 h in the absence of the DNA replication inhibitor AraC (Fig. 2E). O-GlcNAcylation occurred concurrently with A4 accumulation (Fig. 2E). Rifampicin, an inhibitor of virion assembly and core protein processing (31, 32), was used to determine whether assembly was required for O-GlcNAcylation of A4. Cells were infected with vA4-YFP in the presence of rifampicin, and blots of the purified protein were probed with anti-A4 antibody and anti-O-GlcNAc mAbs. The drug did not prevent O-GlcNAcylation of A4, indicating that the modification was independent of core formation (Fig. 2F).

### Evidence that A4 is the sole O-GlcNAc virion protein

**Fig 3:**
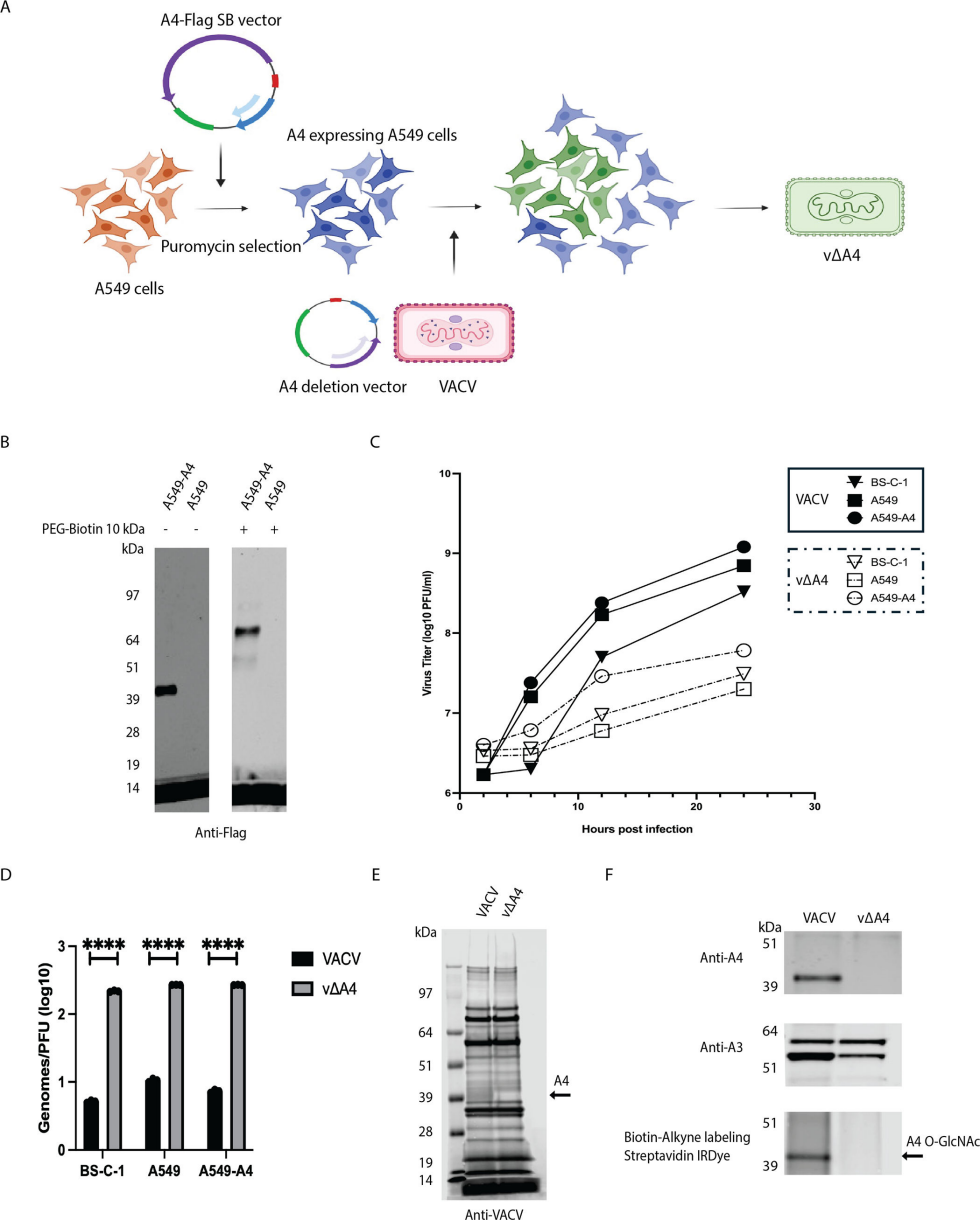
A4 is the only virion O-GlcNAc protein. (**A**) Outline of the scheme for construction of an A549 cell line that stably expresses A4-Flag and creation of vΔA4, a recombinant VACV with GFP replacing A4, by homologous recombination. (**B**) Stable expression and O-GlcNAcylation of A4. A549-A4 and A549 cell lysates were untreated (–) or treated (+) with PEG-Biotin 10 kDa and analyzed by SDS-PAGE. Blots were probed with anti-Flag antibody. (**C**) Effects of A4 deletion on virus yields. BS-C-1, A549, and A549-A4 cells were infected with VACV or vΔA4 at a multiplicity of 1 PFU per cell. One-step virus growth curves were generated by measuring viral titers at the indicated time points using plaque assays on BS-C-1 cells. (**D**) Reduced infectivity of vΔA4. VACV and vΔA4 virions were purified from BS-C-1, A549, and A549-A4 cells. Genomic DNA was determined by digital polymerase chain reaction and plaque-forming units in the respective cell lines. Genomes/PFU ratios are plotted. Data were collected from three independent infections. Bars represent standard deviations. Statistical significance was determined with ANOVA multiple comparison test. (**E**) Protein composition of purified VACV and vΔA4. Proteins extracted from purified VACV or vΔA4 were resolved by SDS-PAGE, and blots were probed with anti-VACV antibodies. The arrow points to the absence of A4. (**F**) Absence of O-GlcNAcylated proteins in purified vΔA4 virions. Proteins from purified VACV and vΔA4 were treated with Biotin-Alkyne and analyzed by SDS-PAGE. Blots were probed with antibodies to A4 and A3 and streptavidin IRDye.

The demonstration that A4 is an O-GlcNAcylated protein of 40 kDa size did not rule out the possibility of another O-GlcNAc protein of similar size. To determine whether additional VACV O-GlcNAc modified proteins of ~40 kDa exist, we constructed an A4 deletion mutant (vΔA4), as depicted in Fig. 3A. The first step was to construct A549 cells stably expressing A4-Flag (A549-A4) for complementation. Western blotting with anti-Flag antibody confirmed the expression of A4 in A549-A4 cells, and labeling with PEG-Biotin 10 kDa demonstrated O-GlcNAcylation (Fig. 3B). The A549-A4 cells were infected with VACV and transfected with a plasmid containing the GFP ORF regulated by a VACV promoter and flanked by sequences upstream and downstream of the A4L ORF to allow replacement of A4 by homologous recombination (Fig. 3A). Virus was isolated from small green fluorescing plaques and clonally purified by repeated plaque isolations. Complete genome sequencing confirmed the absence of the A4L ORF or additional mutations that might contribute to the phenotype of the virus. Previous studies had shown that repression of A4 expression severely reduced the size of virus plaques and reduced the yield of infectious progeny by 1 to 2 logs, although a deletion mutant was not previously isolated (20). Similarly, the yields of vΔA4 were 1 to 2 logs lower than those of wild-type VACV in BS-C-1 and A549 cells. The yield of vΔA4 was less than a log higher in A549-A4 cells compared to control A549 cells, indicating only partial complementation of infectivity (Fig. 3C). Nevertheless, we produced stocks of virus adequate for further experiments. The vΔA4 virus particles purified by sucrose gradient sedimentation had a high DNA-to-plaque-forming units (PFU) ratio compared to wild-type VACV, demonstrating that the particles are defective in infectivity (Fig. 3D). SDS-PAGE analysis suggested that the aberrant particles contained the major virion proteins, except for A4 (Fig. 3E). However, no detectable O-GlcNAc protein was detected by streptavidin IRDye of Biotin-Alkyne-labeled virion proteins from the A4 deletion mutant (Fig. 3F). Our combined analyses involving click chemistry labeling, mass spectrometry, and the A4 deletion mutant strongly support the conclusion that A4 is the predominant—and potentially the sole—O-GlcNAcylated protein within VACV virions.

### Identification of O-GlcNAc-modified amino acids within the A4 protein

**Fig 4:**
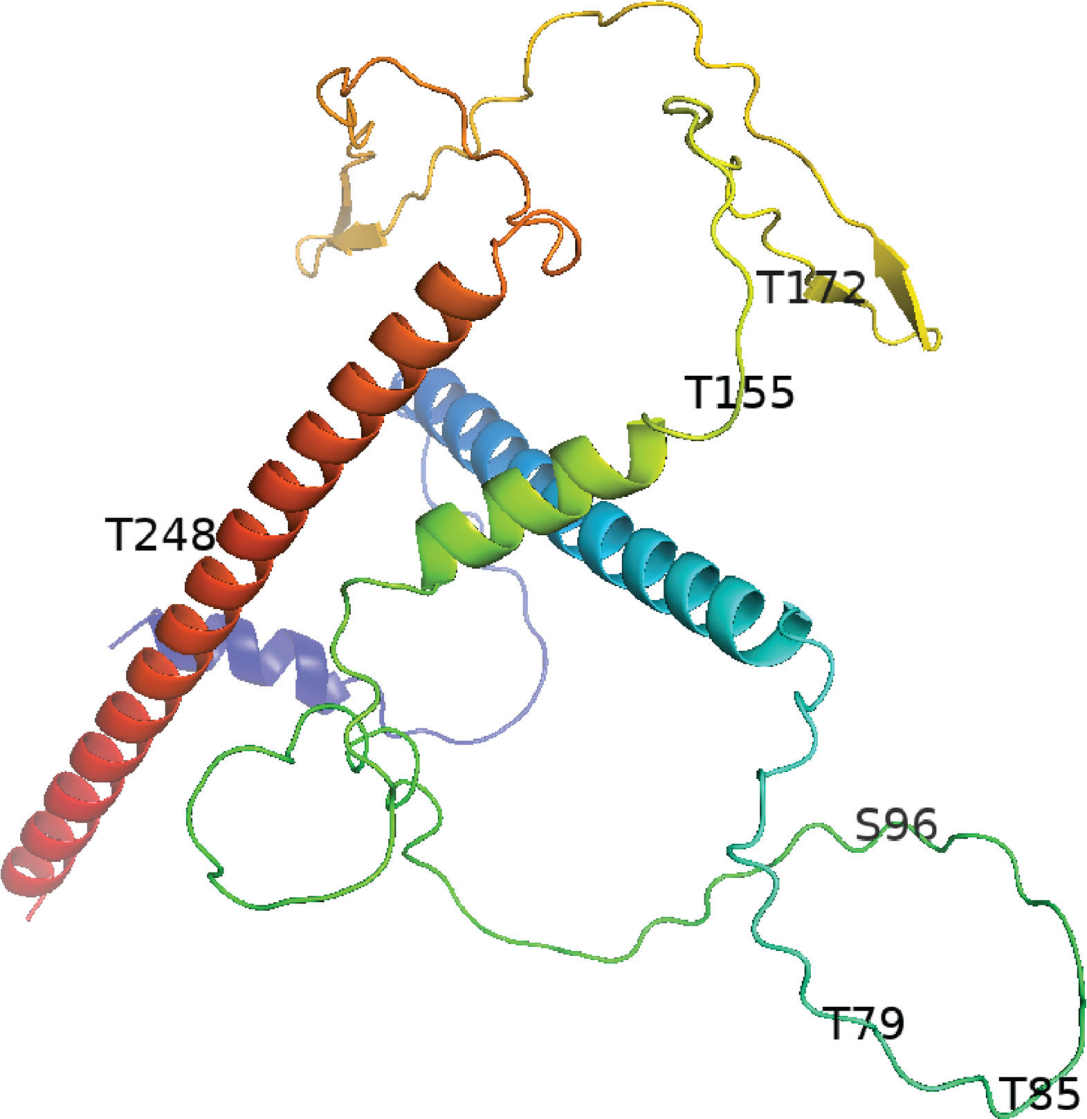
Location of O-GlcNAc modifications within predicted A4 protein structure. The A4 structure largely comprises intrinsically disordered regions, α-helices, and two short antiparallel β-sheets, as predicted by AlphaFold2. Except for T248, all the identified O-GlcNAc modifications are within the disordered regions.

The VACV 281 amino acid A4 protein contains 61 Ser and Thr residues providing multiple potential O-GlcNAc sites. Computational predictions using O-GlcNAcPRED-DL (https://oglcnac.org/pred_dl/) identified up to 35 potential modification sites with varying levels of confidence, underscoring the potential complexity of O-GlcNAcylation on A4. To experimentally investigate the modification sites, A4-YFP was purified from infected cell lysates for subsequent analysis. In order to obtain the maximal coverage of A4, single proteolytic enzymes including trypsin, chymotrypsin, and elastase and a combination of trypsin and α-lytic protease were used to generate peptides for ETD MS (33). Peptides STVPTPK with an O-GlcNAc at Ser96, SKFNKDQKTTTPPSTQPS with an O-GlcNAc at Thr155, CTQQSDGNIS with an O-GlcNAc at Thr172, and DMHQLQAETNDLV with GlcNAc at Thr248 were identified based on the characteristic HexNAc oxonium ion (Table 2). Additionally, the residues Thr79 and Thr85 in the peptide IHITPQPVPTATPAPI had multiple HexNAc and Hex units, suggesting a more complex glycosylation modification. Additional low-frequency O-GlcNAc sites were not excluded. A4 is highly conserved in Orthopoxviruses, and sequence comparisons indicated that the Ser and Thr residues that are modified by O-GlcNAc in VACV are present in other members of the genus. An AlphaFold2 protein prediction model of A4 indicated that it largely comprises intrinsically disordered regions and α-helices (Fig. 4). Except for Thr248, the O-GlcNAc modifications are within the disordered regions of A4.

**TABLE 2:** Modification sites identified by MS analysis^*a*^

Pos.	Sequence	Mods (variable)	Glycans	Fragment type
79	T.IHI**T**[+203.07937]PQPVPTATPAPI.L	T4(OGlycan / 203.0794)	HexNAc (1)	HCD
79	T.IHI**T**[+365.13220]PQPVPTATPAPI.L	T4(OGlycan / 365.1322)	HexNAc (1)Hex (1)	HCD
79	T.IHI**T**[+406.15875]PQPVPTATPAPI.L	T4(OGlycan / 406.1587)	HexNAc (2)	HCD
85	T.IHITPQPVP**T**[+365.13220]ATPAPI.L	T10(OGlycan / 365.1322)	HexNAc (1)Hex (1)	HCD
96	S.**S**[+203.07937]TVPTPK.P	S1(OGlycan / 203.0794)	HexNAc (1)	HCD
155	I.SKFNKDQKT**T**[+203.07937]TPPSTQPS.Q	T10(OGlycan / 203.0794)	HexNAc (1)	HCD
172	T.C[+57.02146]**T**[+203.07937]QQSDGNIS.C	T2(OGlycan / 203.0794)	HexNAc (1)	HCD
248	R.DMHQLQAE**T**[+203.07937]NDLV.T	T9(OGlycan / 203.0794)	HexNAc (1)	HCD

^*a*^ Pos.: position of the modified residue in the peptide sequence. Sequence: amino acid sequence of the peptide with modified T or S in bold and mass changes in brackets [ ]. Mods (variable): type of modification, including glycosylation type and associated mass shift. Glycans: structural information about the glycan attached. HexNAc(n): N-acetylhexosamine, where "n" represents the number of such residues in the glycan. Hex(n): hexose sugars (e.g., glucose, mannose, and galactose), where "n" represents the number of such residues in the glycan. Fragment type: the type of fragmentation used during mass spectrometry (e.g., HCD for higher-energy collisional dissociation and ETD for electron transfer dissociation).

### VACV H3 is not required for O-GlcNAcylation of A4

**Fig 5:**
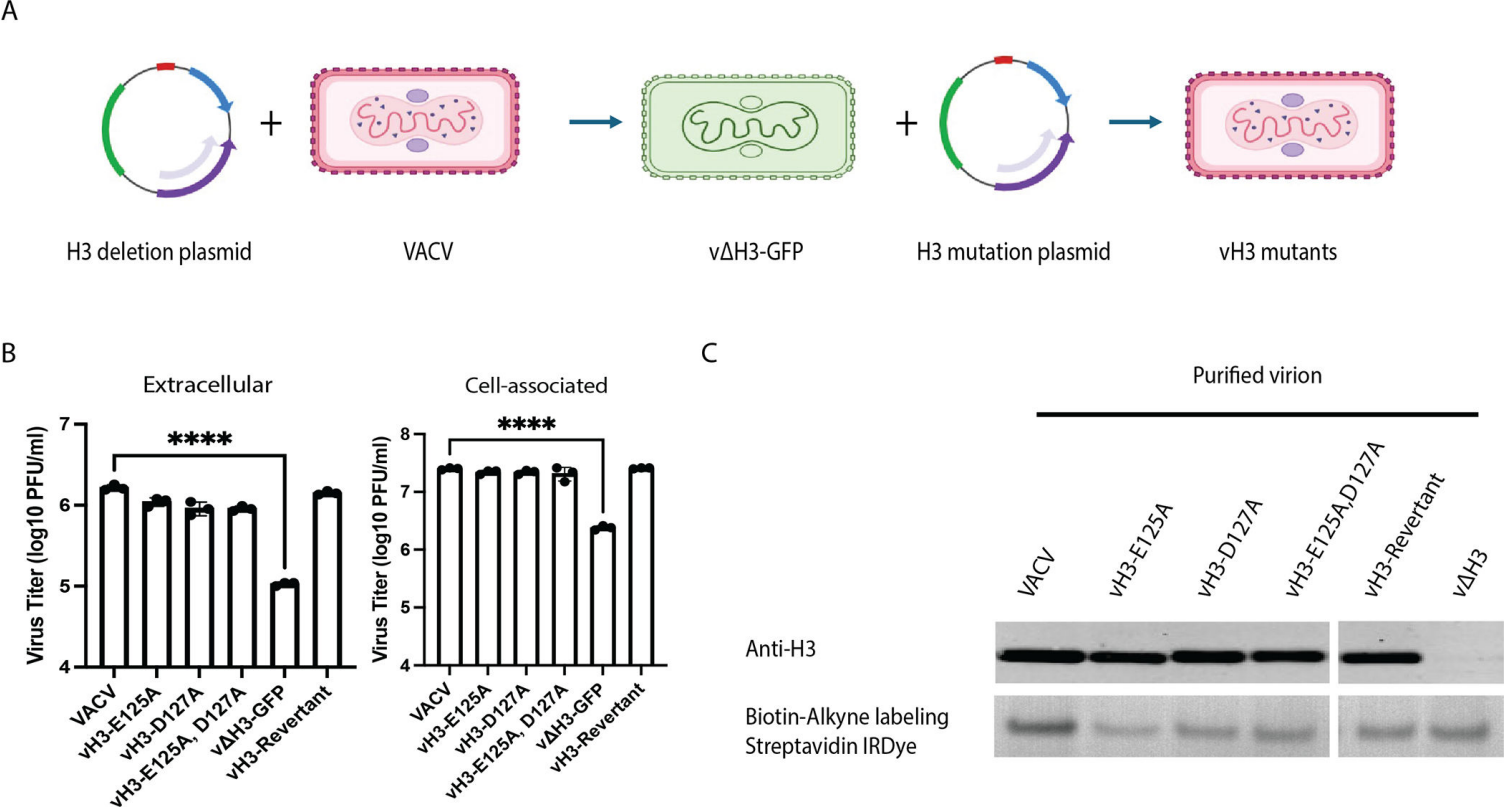
Neither mutations of the putative glycosyltransferase domain of H3 nor deletion of H3 prevent O-GlcNAcylation. (**A**) Scheme depicting the replacement of the H3L ORF by GFP to make vΔH3-GFP and subsequent replacement of GFP with H3L ORF containing single mutations (vH3-E125A and vH3-D127A), double mutations (vH3-E125A and D127A), and a revertant control. (**B**) Effects of mutations and deletion of H3 on virus yields. HeLa cells were infected with 1 PFU/cell of VACV, H3 point mutants, vΔH3-GFP, or revertant for 24 h. Virus titers in medium and cell lysates were determined by plaque assay. Data were collected from three independent infections. Bars represent standard deviations. Statistical significance was determined with ANOVA multiple comparison test. (**C**) Effects of H3 mutations on O-GlcNAc. Proteins from purified VACV and the indicated H3 mutants were conjugated to Biotin-Alkyne and analyzed by SDS-PAGE, followed by immunoblotting with anti-H3 antibody and streptavidin-IRDye.

The ability of the cellular OGT to modify A4 was demonstrated in uninfected A549 cells stably expressing A4-Flag (Fig. 3B). However, poxvirus proteins are synthesized in cytoplasmic factories where they are assembled into virus particles. It remained possible that a viral enzyme mediates O-GlcNAcylation during infection. In this context, a previous structural study predicted a glycosyltransferase active site including Glu125 and Asp127 of the VACV H3 protein and additionally showed evidence for binding of UDP-glucose to H3 (34). H3 is expressed late in infection, localizes to the MV membrane, and participates in cell attachment and virion assembly (35). To determine if H3 is required for A4 O-GlcNAcylation during virus infection, we constructed viruses (vH3-E125A and vH3-D127A) with mutations at the putative transferase active site, a virus with mutations of both amino acids (vH3-E125A and D127A), a deletion mutant (vΔH3-GFP) in which H3 was replaced by GFP, and a revertant of the latter virus in which H3 was restored, as outlined in Fig. 5A. In brief, the H3L ORF was first replaced with the GFP ORF, and small fluorescent green plaques were isolated. Next, the GFP ORF was replaced with H3L containing point mutations or intact H3L to make a revertant, and non-fluorescent plaques were isolated. The mutations were confirmed by PCR and sequencing. Although the yields of intracellular and extracellular virus were reduced by about a log in cells infected with the deletion mutant, neither the individual nor combined point mutations had much effect (Fig. 5B). However, click chemistry analyses detected the 40 kDa O-GlcNAc modified protein in the MVs of each active site mutant as well as in an H3-deletion mutant (Fig. 5C). As no other candidate viral OGT existed, we considered it likely that the cellular enzyme catalyzed O-GlcNAcylation of A4 during infection.

### O-GlcNAcylation of A4 is reduced by inhibition of OGT

**Fig 6:**
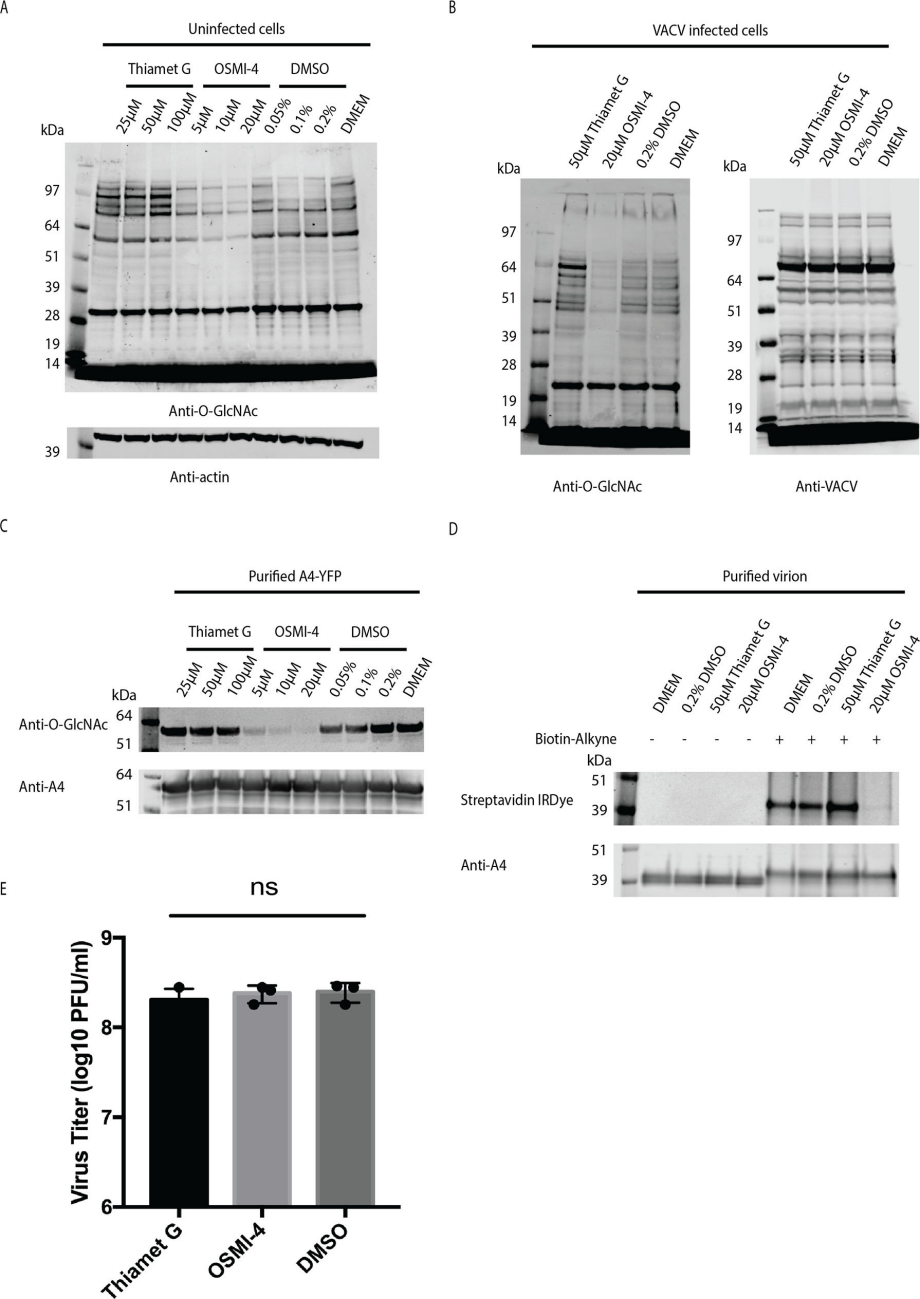
Inhibition of A4 O-GlcNAcylation by OSMI-4. (**A**) Effects of OGA and OGT inhibitors on O-GlcNAcylation of uninfected cell proteins. A549 cells were treated with indicated concentrations of Thiamet G, OSMI-4, DMSO, or with Dulbecco’s minimal essential medium. Lysates were analyzed by SDS-PAGE, and blots were probed with anti-O-GlcNAc mAbs and anti-actin antibody. (**B**) Effects of OGA and OGT inhibitors on VACV-infected cells. Inhibitors were added to A549 cells, and 2 h later, they were infected for 24 h with 3 PFU/cell of VACV. Lysates were analyzed by SDS-PAGE, and blots were probed with anti-O-GlcNAc mAbs or anti-VACV antibodies. (**C**) Effects of OGA and OGT inhibitors on O-GlcNAcylation of A4. A549 cells were treated with inhibitors for 2 h and infected with 3 PFU/cell vA4-YFP for 24 h. A4-YFP was purified from cell lysates with GFP-Trap magnetic agarose beads and analyzed by SDS-PAGE. Blots were probed with anti-O-GlcNAc mAbs and anti-A4 antibody. (**D**) Effects of OGA and OGT inhibitors on O-GlcNAcylation of virion-associated A4. Virions were purified from cells treated and infected with VACV as in panel B, and proteins were untreated (–) or conjugated to Biotin-Alkyne (+) and analyzed by SDS-PAGE. Blots were probed with streptavidin IRDye or anti-A4 antibody. (**E**) Effects of OGA and OGT inhibitors on virus replication. A549 cells that had been treated with 50 µM Thiamet G, 20 µM OMSI-4, or 0.2% DMSO were infected with 1 PFU/cell of VACV in triplicate for 24 h. Virus yields were determined by plaque assay on BS-C-1 cells. Bars represent standard deviation. ns, no significant difference determined by the ANOVA multiple comparison test.

Experiments were carried out using Thiamet G and OSMI-4, inhibitors of OGA and OGT, respectively (36, 37), to determine whether these cellular enzymes regulate O-GlcNAcylation of A4 expressed by VACV. In a pilot experiment, increasing amounts of the inhibitors were added to uninfected HeLa cells to determine the optimal concentrations compared to DMSO used for dissolving OSMI-4. Within a 24 h period, no significant differences in cell viability were detected by MTT assay at concentrations of up to 100 µM Thiamet G and 20 µM OSMI-4. Next, HeLa cells were treated for 24 h with 25, 50, or 100 µM Thiamet G or 5, 10, or 20 µM OSMI-4 or 0.05%, 0.1%, or 0.2% DMSO. Cell lysates were analyzed by SDS-PAGE, and blots were probed with anti-O-GlcNAc mAbs. Cellular O-GlcNAcylated proteins were increased by the OGA inhibitor Thiamet G, while the OGT inhibitor OSMI-4 caused a reduction (Fig. 6A). Based on these data, cells were pretreated with 50 µM Thiamet G, 20 µM OSMI-4, or 0.2% DMSO for 2 h and then infected with VACV, with the inhibitors and DMSO maintained throughout the 24 h infection period. SDS-PAGE blots probed with anti-O-GlcNAc mAbs confirmed that the major O-GlcNAcylated host proteins in infected cells were diminished following OSMI-4 treatment (Fig. 6B). However, replicate blots probed with antibody to VACV indicated that viral protein synthesis was unperturbed by the inhibitors (Fig. 6B).

The effects of OGT and OGA inhibitors on A4 O-GlcNAcylation were determined by adding the drugs to cells prior to infection with vA4-YFP and purifying the A4-YFP fusion protein (Fig. 6C). The O-GlcNAcylation level of A4-YFP increased slightly with Thiamet G and was greatly decreased by OSMI-4, with only a faint band of O-GlcNAcylated A4-YFP still detectable at 20 µM OSMI-4. We considered the possibility that small amounts of residual O-GlcNAc-modified A4 might be selectively incorporated into virus particles. To evaluate this possibility, VACV was purified following treatment of infected cells with 50 µM Thiamet G, 20 µM OSMI-4, 0.2% DMSO, or culture medium. Virion proteins were conjugated to Biotin-Alkyne and analyzed by SDS-PAGE. Blots were probed with streptavidin IRDye and anti-A4 Ab. Only a faint band was detected with streptavidin in virions made in the presence of OSMI-4, whereas none of the treatments influenced the abundance of A4 packaged in virions (Fig. 6D). Furthermore, even though there was a profound inhibition of the O-GlcNAc modification of A4 by OSMI-4, there was no reduction in virus yield (Fig. 6E).

### VACV infectivity was not reduced by degradation of cellular OGT and absence of A4 O-GlcNAcylation

**Fig 7:**
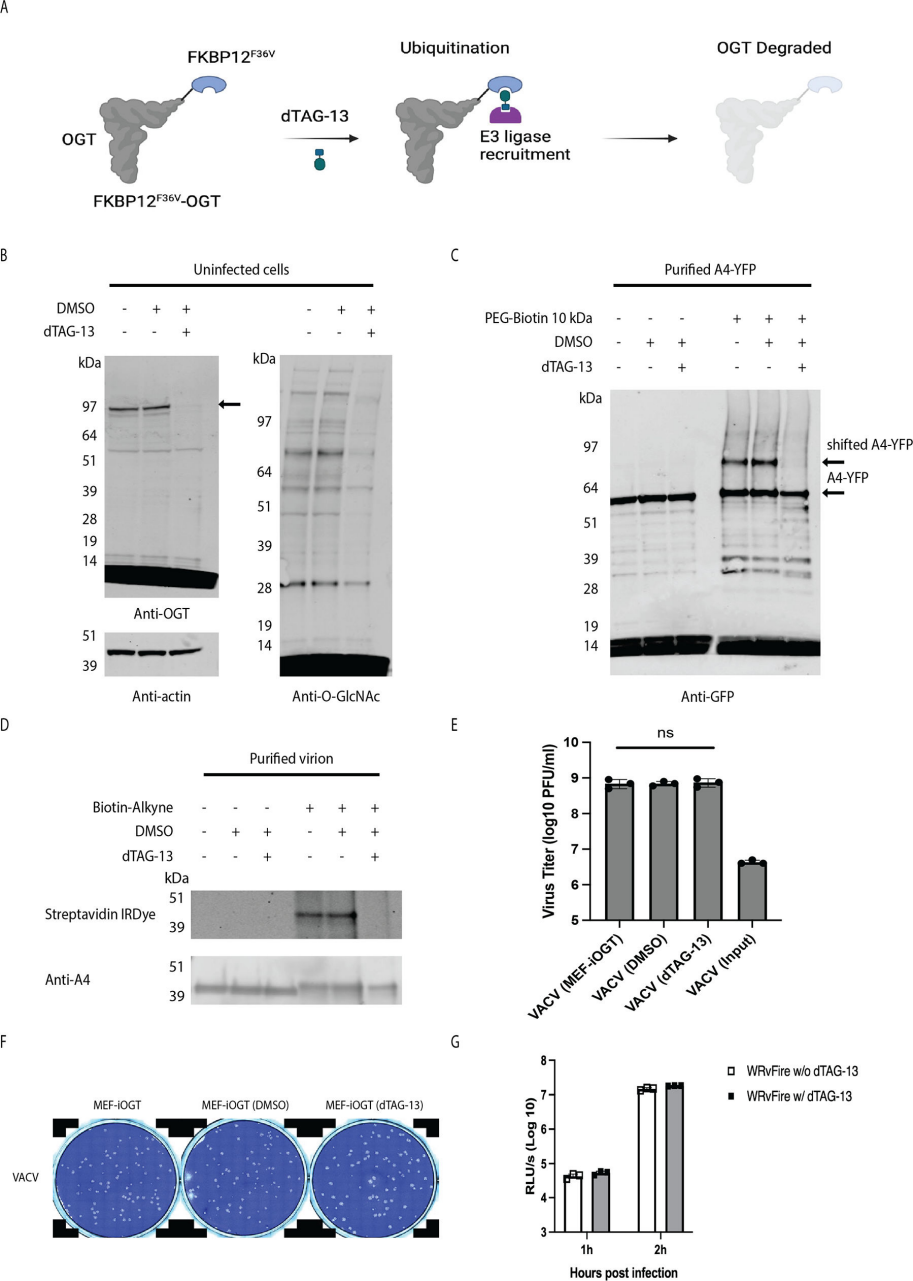
O-GlcNAcylation of A4 is not required for VACV infectivity. (**A**) Scheme for induced degradation of OTG. The heterobifunctional dTAG-13 molecule binds to the modified OGT of MEF FKBP12^F36V^-OGT (MEF-iOGT) cells, leading to the recruitment of the E3 ligase cereblon, which facilitates ubiquitination and subsequent proteasomal degradation of OGT. (**B**) Degradation of OGT and decreased O-GlcNAc proteins in uninfected MEF-iOGT cells. Lysates of MEF-iOGT cells that had been incubated with dTAG-13 or DMSO for 2 h were analyzed by SDS-PAGE. Blots were probed with anti-OGT, anti-actin, and anti-O-GlcNAc antibodies. (**C**) Effect of degradation of OGT on A4 O-GlcNAcylation. MEF-iOGT cells were pretreated with DMSO or dTAG-13 for 2 h and then infected with vA4-YFP in the continued presence of DMSO or dTAG-13. After 24 h, the A4-YFP protein was isolated using GFP-Trap magnetic agarose beads and conjugated with PEG-Biotin 10 kDa. Proteins were analyzed by SDS-PAGE and blots probed with anti-GFP Ab. (**D**) Analysis of O-GlcNAc in purified virions. MEF-iOGT cells were pretreated with DMSO or dTAG-13 for 2 h and infected with VACV in continued presence of DMSO or dTAG-13. After 24 h, virions were purified and proteins reacted with Biotin-Alkyne and analyzed by SDS-PAGE. Blots were probed with anti-A4 Ab and streptavidin IRDye. (**E**) Effect of OGT degradation on virus yields. MEF-iOGT cells were untreated (VACV (MEF-iOGT)) or treated with DMSO (VACV (DMSO)) or dTAG-13 (VACV (dTAG-13)) for 2 h and then infected with VACV in the continued absence or presence of DMSO or dTAG-13. After 24 h, cells were lysed and virus titers determined by plaque assay on BS-C-1 cells. The input titer was determined by harvesting cells following 1 h virus adsorption. Data were collected from three independent infections. Bars represent standard deviations. Statistical significance was determined with ANOVA multiple comparison test. ns indicates *P* > 0.05. (**F**) Degradation of OGT slightly increases plaque size. MEF-iOGT cell monolayers were infected with VACV in the presence of DMSO (MEF-iOGT (DMSO)) or dTAG-13 (MEF-iOGT (dTAG-13)), as indicated, with untreated cells (MEF-iOGT (MEF-iOGT)) as the control. After 48 h, plates were stained with crystal violet. (**G**) Loss of A4 O-GlcNAcylation does not impair VACV entry. Purified WRvFire virions derived from MEF-iOGT cells treated with or without dTAG-13 were used to infect cells at a multiplicity of 3 PFU per cell. Viral entry was assessed by measuring luciferase activity at the indicated time post-infection.

In order to deplete OGT, which is essential for cell proliferation, Levine and co-workers (38) constructed a mouse embryonic fibroblast cell line (MEF-ΔOGT-FKBP12^F36V^-OGT, abbreviated MEF-iOGT) in which the endogenous OGT was replaced with a degron-tagged OGT. As depicted in Fig. 7A, degradation of FKBP12^F36V^-OGT was accomplished by incubation with dTAG-13, a heterobifunctional degrader molecule that targets the fusion protein for E3 ligase cereblon-mediated ubiquitination, leading to proteasomal degradation (39). Upon dTAG-13 treatment of MEF-iOGT cells for 2 h, the OGT was below the detection level, and cellular O-GlcNAcylated proteins were reduced, as determined by immunoblotting (Fig. 7B). Having confirmed the rapid degradation of OGT, MEF-iOGT cells were pretreated with dTAG-13 for 2 h and then infected with VACV expressing A4-YFP for 24 h. A4-YFP was purified from cell lysates and evaluated for the presence of O-GlcNAc by mass tagging with the PEG-Biotin 10 kDa tag. The absence of a shifted A4-YFP band suggested near-total inhibition of O-GlcNAcylation (Fig. 7C). Moreover, Biotin-Alkyne click chemistry labeling of VACV purified from dTAG-13-treated MEF-iOGT cells yielded no visible O-GlcNAc band (Fig. 7D). We attribute the total or near-total inhibition of A4 O-GlcNAcylation to the addition of dTAG-13 and degradation of OGT prior to VACV infection.

To determine the effect of A4 O-GlcNAcylation inhibition on viral replication, MEF-iOGT cells pretreated with dTAG-13 or DMSO for 2 h were infected with VACV at a multiplicity of 1. Viral yields from DMSO- and dTAG-13-treated MEF-iOGT cells were equivalent after one (Fig. 7E) or three serial passages. Interestingly, plaques were slightly larger in size in MEF-iOGT cells that were treated with dTAG-13 compared to untreated or DMSO-treated cells (Fig. 7F), possibly reflecting the inhibition of cellular protein O-GlcNAcylation, which could impact immune defense mechanisms. To measure the influence of O-GlcNac modification on viral entry and early gene expression, we used a recombinant VACV (WRvFire) that expresses Firefly luciferase under a viral early promoter. WRvFire was purified from MEF-iOGT cells that had been pretreated with or without dTAG-13 and used to infect HeLa cells. After 1 and 2 h, the cells were lysed and luciferase activity measured. However, no difference was found (Fig. 7G). Thus, by using two different methods to knock down O-GlcNAcylation of A4, the virus retained infectivity in cultured cells.

## DISCUSSION

This study was undertaken as a follow-up to the intriguing discovery, made more than 50 years ago, of a 40 kDa glucosamine-labeled VACV protein (5). At the time, the finding was novel due to the small size of the carbohydrate and absence of other sugars but largely unappreciated. It was not until 1983, 13 years later, that the first O-GlcNAc proteins were characterized (7). The delay in recognizing the O-GlcNAc modification can be attributed to multiple factors including small size with minimal effect on electrophoretic mobility, weak binding to lectins, presence in sub-stoichiometric amounts, lability on MS analysis, and removal by cellular hydrolases (8). The main objectives of the current study were to confirm the presence of an O-GlcNAc modification in VACV virions, identify the modified protein, determine the modification sites, identify the enzyme responsible for biosynthesis, and ascertain a function for the modification. We were successful in all but the last goal.

We used improved methods of detecting O-GlcNAc modifications, in particular chemoenzymatic click chemistry labeling, specific mAbs, and more sophisticated mass spectrometry techniques (8). Click chemistry followed by SDS-PAGE allowed us to achieve our first goal: the detection of a single 40 kDa O-GlcNAc protein in purified VACV virions. Identification of the modified proteins was more difficult and required size enrichment of click chemistry-labeled proteins, mass spectrometry, and most importantly, a click chemistry mass tagging method (40) that unambiguously identified the A4 protein. In our adaptation of the method, a mass of 10 kDa was expected for each O-GlcNAc moiety. The major band exhibited an increase of 20 kDa, and minor bands were increased by 60 kDa or more, indicating multiple sites, at least some of which were incompletely modified. Additionally, the homologous CPXV protein was modified with O-GlcNAc. The absence of a detectable O-GlcNAc modification of aberrant virus particles that formed when the A4 gene was deleted from VACV confirmed the identity of the modified protein and suggested that there are no other O-GlcNAc proteins associated with virions. Collectively, these analyses—including selective mass tagging and examination of the A4 deletion mutant—robustly support our conclusion that A4 is the predominant, and potentially sole, O-GlcNAcylated protein within VACV virions. A4 is a highly conserved core protein that is required for virion assembly (41) and interacts with other core proteins (30, 42). Whether some VACV nonstructural proteins are O-GlcNAcylated was not determined due to the numerous modified cellular proteins in infected cell lysates.

During conventional mass spectroscopy, the O-GlcNAc residue may be lost by ionization, and the signal can be overwhelmed by the presence of unmodified peptides. The development of ETD mass spectrometry provided a breakthrough for O-GlcNAc protein site assignments (43). The application of this technique to A4 was hindered in part by the amino acid sequence, which resulted in very large peptides produced by trypsin and some other proteinases. However, with a mixture of proteinases, coverage of most of the protein was obtained, leading to the detection of at least four O-GlcNAc attachment sites, consistent with evidence for multiple sites from our mass-tagging data. A4 is predicted to have three intrinsically disordered regions with an enrichment of polar amino acids making up about one-third of the protein length. Three of the four O-GlcNAc residues were localized to disordered regions. Disordered regions are also a common site of O-GlcNAc modification of cellular proteins (12).

The next question that we addressed was whether viral or cellular glycosyltransferases are responsible for O-GlcNAcylation of A4. The possibility of a viral enzyme was intriguing because poxviruses encode DNA polymerase, RNA polymerase, kinases, phosphatases, and even their own cytoplasmic redox system (1). In addition, viral structural proteins are synthesized and virions assembled in specialized viral factories. The VACV H3 protein was a candidate O-GlcNAc transferase as structural studies revealed a putative glycosyltransferase domain and biochemical studies demonstrated binding to UDP-glucose (34). Nevertheless, neither mutations of the putative active site of H3 nor deletion of the H3 gene abrogated O-GlcNAc modification. As VACV has no other protein that resembles a glycosyltransferase, it seemed most likely that the cellular OGT adds O-GlcNAc residues to A4. Initially, we demonstrated that A4-Flag and -YFP fusion proteins were modified by the addition of O-GlcNAc residues upon transfection into uninfected cells. While this experiment demonstrated that A4 sequences are recognized as substrates by OGT, it was necessary to prove this enzyme regulates O-GlcNAcylation of A4 during a virus infection. To accomplish this, we first used an inhibitor of OGT (36). Addition of OSMI-4 to cells greatly decreased O-GlcNAc modification of A4 without an effect on the expression of VACV proteins or incorporation of unmodified A4 into virus particles. As further proof of the role of OGT, we used the dTAG degron system for degradation of OGT (39) prior to VACV infection. Under these conditions, O-GlcNAcylation of A4 was undetectable. Nevertheless, the absence of O-GlcNAc had no effect on VACV replication or virion infectivity, bringing us to the final question of the role of A4 O-GlcNAcylation.

Additional experiments, including sensitive luciferase-based viral entry-fusion assays using the WRvFire reporter virus, revealed no significant differences in viral entry or infection kinetics attributable to the presence or absence of this modification under standard *in vitro* conditions. These results indicate that A4 O-GlcNAcylation is not essential for fundamental steps of VACV replication within typical cell culture models. Unfortunately, A4 has not been resolved by cryo-electron microscopy because of its intrinsically disordered regions (44), precluding any attempts to determine whether O-GlcNAcylation perturbs A4 structure. Although A4 has only been shown to have a role in virus morphogenesis and binding to the major core protein (30, 41), we cannot exclude the possibility of additional unrecognized functions for A4 that might confer more subtle effects for A4 O-GlcNAcylation under alternative or more physiologically relevant conditions. For instance, in complex *in vivo* environments or during specific host immune interactions, the modification might influence viral pathogenesis, infectivity, or immune evasion mechanisms in ways not apparent in short-term cell culture assays. However, as O-GlcNAcylation of cellular proteins is essential, it is not currently possible to confirm or extend our *in vitro* experiments in an animal model lacking OGT.

An additional consideration is the potential promiscuity of cellular OGT, which preferentially targets accessible serine/threonine residues within intrinsically disordered protein regions, as observed with A4. Although thousands of cellular proteins undergo O-GlcNAc modification, in relatively few cases have clearly defined functional roles been shown. Thus, A4 O-GlcNAcylation might represent a bystander modification without a critical functional impact, or it may subtly modulate interactions with host cell signaling pathways or protein complexes involved in virion assembly or release.

## MATERIALS AND METHODS

### Cells and virus

HeLa cells and African green monkey kidney cells (BS-C-1) were cultured in Eagle’s Minimum Essential Medium (EMEM) supplemented with 10% fetal bovine serum (FBS). Human embryonic kidney cells (293T) were grown in Dulbecco’s modified Eagle’s medium (DMEM) with 10% FBS, and human lung epithelium A549 cells were cultured in DMEM/F12 medium supplemented with 10% FBS. MEF-iOGT cells were generously provided by Suzanne Walker (38) and grown in DMEM supplemented with 15% FBS. VACV strain WR was propagated in HeLa cells, and all recombinant viruses were derived from the WR strain and constructions approved by the NIH Institutional Biosafety Committee.

### Virus purification

VACV and recombinant viruses were grown in HeLa cells and purified by ultracentrifugation through a 36% (wt/vol) sucrose cushion, followed by banding through 24% to 40% sucrose gradients. After resuspension in 1 mM Tris-Cl, pH 9.0, virus aliquots were stored at −80 ℃. Infectivity in BS-C-1 cells was determined by plaque assay (45).

### Protein extraction

Cells were lysed using RIPA buffer (10 mM Tris-Cl pH 7.5, 150 mM NaCl, 0.5 mM EDTA, 0.1% SDS, 1% Triton X-100, and 1% deoxycholate) supplemented with protease and phosphatase inhibitor cocktail (Sigma) and DNase I (ThermoFisher) on ice. Proteins were extracted from purified virions with 2% SDS in 100 mM Tris, pH 8, supplemented with protease and phosphatase inhibitors, and boiled for 5 min. Alternatively, virions were first incubated with 0.5% NP-40 in 50 mM Tris, pH 8 to extract membrane proteins, and post-extraction, pellets were further treated with 2% SDS in 100 mM Tris, pH 8. Protein concentrations were determined using the Qubit Protein BR Assay kit (Invitrogen).

### Immunoblotting

Solubilized proteins were separated by SDS-PAGE (4%–12% gels), followed by Coomassie blue staining or transfer to nitrocellulose membranes (iBlot 2 NC stacks, Invitrogen). Membranes were blocked with 5% nonfat milk solution and hybridized with primary antibodies overnight at 4°C. After washing, membranes were probed with IRDye-conjugated secondary antibodies (Li-COR) for visualization. Primary antibodies included anti-O-GlcNAc MultiMab mix (Cell Signaling Technology, #82332), IRDye 800CW Streptavidin (Li-COR), anti-GFP polyclonal antibody (Invitrogen, A11122), anti-Flag M2 antibody (Sigma, F1804), anti-A4 antibody (46), anti-D8 antibody (47), anti-H3 antibody (48), anti-L3 antibody (49), anti-A3 antibody (50).

### O-GlcNAc click chemistry labeling with Biotin-Alkyne

For chemoenzymatic labeling of O-GlcNAcylated proteins, the Click-iT O-GlcNAc Enzymatic Labeling System (GalT1 (Y289L) mutant) and the Click-iT Glycoprotein detection kit (Biotin Alkyne) were used according to the manufacturer’s instructions (Invitrogen). Briefly, cell lysates and purified virus lysates were extracted using 2% SDS with 100 mM Tris pH 8, supplemented with protease inhibitor and phosphatase inhibitor cocktails (Sigma), and boiled for 5 min. Subsequently, the proteins were chemoenzymatically labeled by incubating with GalT1 (Y289L) and UDP-GalNAz, co-incubated with PNGase F (10 U/µl) overnight at 4 ℃, followed by CuAAC addition of biotin at room temperature for 2 h. The labeled proteins were detected by probing immunoblots with IRDye 800CW Streptavidin (Li-COR). Alternatively, for direct visualization of azide-labeled O-GlcNAc proteins on SDS-PAGE gels, the azide-modified proteins were treated with IRDye 800CW Alkyne instead of the Biotin-Alkyne, following the same CuAAC process.

### O-GlcNAc click chemistry labeling with PEG-Biotin 10 kDa

To identify the O-GlcNAc protein in purified VACV virions, SPAAC chemistry methods were followed to add PEG-Biotin 10 kDa (Biopharma PEG) to azide-labeled O-GlcNAc proteins (26). Briefly, purified virus was solubilized with 2% SDS with 100 mM Tris pH 8 supplemented with protease inhibitor and phosphatase inhibitor cocktails (Sigma) at 100°C for 5 min. Subsequently, the proteins were chemoenzymatically labeled by incubating with GalT1 (Y289L) and UDP-GalNAz and PNGase F (10 U/µL) overnight at 4°C and then incubated with PEG-Biotin 10 kDa (1 mM in DMSO) at room temperature overnight. The labeled proteins were analyzed using VACV-specific protein antibodies by immunoblotting.

### Construction of recombinant plasmids

To construct vA4-YFP, a recombinant PCR product was created containing the 5′ portion of the A4L ORF appended to the YFP ORF directly after the start codon of A4L and flanked by portions of the A3L and the A5R ORFs with In-Fusion flanking sites at both termini by overlap extension PCR using VACV strain WR genomic DNA as the template. The DNA was inserted into the pUC19 linearized vector through the In-Fusion HD cloning kit (Takara Bio USA) according to the manufacturer’s instructions.

The A4L gene knockout plasmid was constructed by ligating a portion of the A3L ORF flanked by the In-Fusion flanking site and a segment of the A5R ORF flanked with the In-Fusion flanking site into the pUC19 linearized vector, creating a construct containing the GFP ORF, which is under the regulatory control of the VACV P11 promoter, and flanked by A3L and A5R ORF segments. Similar procedures were used to generate the A4-YFP expression plasmids, in which the 5′ of A4L ORF was appended to the YFP ORF directly after the start codon of A4L and regulated by the CMV promoter in the pcDNA 3.1 + plasmid (Addgene). The A4L ORF of VACV strain WR was modified by mammalian codon optimization for transfection of A4-YFP-expressing plasmids, with Lipofectamine 2000 reagent used (ThermoFisher) in 293T cells.

To construct an H3 deletion virus (vΔH3-GFP), a recombinant PCR product containing a portion of the H2R ORF flanked by the In-Fusion flanking site and a segment of the H4L ORF flanked with In-Fusion flanking site was created. The DNA was inserted into the pUC19 linearized vector, creating a construct containing the GFP ORF, which is under the regulatory control of the P11 promoter, and flanked by H2R and H4L ORF segments via the In-Fusion HD cloning kit according to the manufacturer’s instructions. A similar procedure was used to generate the H3 revertant plasmid in which the GFP ORF was replaced by the wild-type H3L ORF. H3 variants, H3-E125A, H3-D127A, and H3-E125A, D127A were generated by using Q5 Site-Directed Mutagenesis (New England Biolabs) with the H3 revertant sequence in the pUC19 vector. All constructs were confirmed by DNA sequencing.

### A4 expressing A549 cell line

To rescue the A4L gene knock-out VACV (vΔA4), A4 expressing A549 cell lines were generated using the Sleeping Beauty transposon system (51). Briefly, the A4L ORF of VACV strain WR was modified by mammalian codon optimization and generated an additional N-terminal FLAG-tag as well as the SfiI-restriction sites at both ends by overlapping PCR. Correct products were cloned into the psBtet-Pur (plasmid #60507). The A549 cells were transfected with a transposase-encoding plasmid (SB100x) and the Sleeping Beauty construct. Appropriate selection was carried out with puromycin. A total of three rounds of selection were performed, each with a recovery phase lasting two doublings per cell line. The A4 expression was induced by doxycycline and confirmed by the anti-Flag tag and anti-A4 antibodies immunoblotting.

### Construction of recombinant VACV

To generate a VACV that expresses A4 fused to YFP, the recombinant plasmid was linearized by SacI and HindIII and transfected into BS-C-1 cells infected with wild-type VACV strain WR using the Lipofectamine 2000 reagent. Diluted lysates were applied to BS-C-1 monolayers, and plaques containing recombinant virus were detected by fluorescence microscopy. Virus was recovered from fluorescent plaques, and the procedure was repeated until the virus was clonally pure. The recombination and deletion of the genes were confirmed by PCR, DNA sequencing, and immunoblotting. A similar procedure was followed to generate the A4 deletion virus on A4-expressing A549 cells. Virus was recovered from fluorescent plaques, and the procedure was repeated until the virus was clonally pure. The recombination and deletion of the genes were confirmed by PCR, DNA sequencing, and immunoblotting.

To generate a recombinant VACV H3 deletion virus, a recombinant plasmid was linearized by SacI and HindIII digestion and transfected with Lipofectamine 2000 reagent into BS-C-1 cells infected with wild-type VACV strain WR, allowing homologous recombination to occur. Diluted lysates were applied to BS-C-1 monolayers, and plaques containing recombinant virus were detected by fluorescence microscopy. Virus was recovered from fluorescent plaques, and the procedure was repeated until the virus was clonally pure. The recombination and deletion of the genes were confirmed by PCR, DNA sequencing, and immunoblotting. The recombinant VACV H3 variant viruses were derived from the H3 deletion virus by homologous recombination, following a similar protocol to select non-fluorescent plaques on BS-C-1 cells.

### Affinity protein purification

Cells were washed twice with cold phosphate-buffered saline (PBS) on ice, harvested by scraping, and lysed in RIPA buffer containing protease inhibitor and phosphatase inhibitors (Sigma) and DNase I (ThermoFisher) on wet ice for 30 min with frequent agitation. Lysates were centrifuged for 10 min at 20,000 × *g* at 4°C, and the supernatant was incubated with GFP-Trap magnetic agarose beads (ChromoTek) at 4°C for 2 h. After washing three times with 10 mM Tris/Cl pH 7.5, 150 mM NaCl, 0.05% NP-40, 0.5 mM EDTA, the bound proteins were eluted with 200 mM glycine pH 2.5 and immediately neutralized with 1 M Tris pH 10.4. Protein concentrations were determined by the Qubit Protein BR Assay kit (Invitrogen).

### Time course of O-GlcNAcylation of A4

To determine the synthesis and accumulation of A4 O-GlcNAc, HeLa cells were infected with 3 PFU/cell of vA4-YFP and washed three times after 1 h absorption. Cell lysates were prepared at 2, 6, 12, 18, and 24 h post-infection. The lysates collected after 1 h absorption served as the 0 h post-infection control. Cytosine arabinoside (AraC, 44 µg/mL) was used to inhibit DNA replication and prevent intermediate and late protein synthesis for 24 h. The accumulation of A4 O-GlcNAc was determined on the A4-YFP protein after purification with GFP-Trap magnetic agarose (ChromoTek).

### Effect of viral assembly inhibitor on A4 O-GlcNAc

To investigate the influence of viral assembly on the O-GlcNAc modification of the A4 protein, HeLa cells were infected with 3 PFU of vA4-YFP per cell. After 1 h absorption, cells were washed three times and incubated with or without rifampicin (100 µg/mL) for 2 and 18 h. The A4 protein was then isolated using GFP-Trap magnetic agarose beads and analyzed for synthesis and O-GlcNAc levels through immunoblotting.

### One-step virus growth

BS-C-1, A549, and doxycycline-induced A549-A4 cells were infected with VACV strain WR or vΔA4 virus with 1 PFU of virus/cell. Following adsorption for 1 hour at 37°C, cells were washed three times and then incubated for various time periods. Virus was harvested by subjecting cells to three freeze-thaw cycles, stored at −80°C, and viral titers were subsequently determined by plaque assay on BS-C-1 cell monolayers overlaid with 0.5% methylcellulose in complete medium.

### Quantification of viral genome copies by droplet digital PCR (ddPCR)

The wild-type VACV and A4 deletion viruses were purified by sucrose cushion and gradient, and the virus titer was determined by plaque assay on BS-C-1 cells. The viral DNA was extracted using a DNeasy blood and tissue kit (QIAGEN) according to the manufacturer’s protocols and treated with benzonase. The DNA was serially diluted and analyzed with gene-specific primers and QX200 ddPCR EvaGreen Supermix (Bio-Rad) by ddPCR following the protocol described previously (52). After 40 reaction cycles, the droplets were digitally analyzed with a droplet reader (Bio-Rad), and absolute DNA copy numbers were determined. Viral genome copies were measured by primers targeting the E11 gene (53).

### LC-MS-MS analysis of O-GlcNAc-enriched proteins

LC-MS-MS was carried out at the NIAID core facility. Samples were injected into a Thermo Orbitrap Fusion operated with an in-line Thermo nLC 1200 and an EASY-Spray ion source. In-line chromatography utilized a 2 cm PepMap 100 C18 trap column (3 µm, Φ 75 µm) and a 25 cm Easy-Spray PepMap 100 C18 analytical column (2 µm, Φ 75 µm). Peptides were eluted over the course of an 80 min gradient from solvent A (water +0.1% formic acid) to 50% solvent B (80/20 acetonitrile/water +0.1% formic acid). This was followed by a 5 minute rapid increase to 100% solvent B, where it was held for a subsequent 5 min. Instrument acquisitions were operated at a 120,000 resolution (*m/z* 200) with a scan range of 400–2,000 *m/z*. CID and HCD fragmentation were performed utilizing a targeted fragment list of characteristic ions to prioritize the identification of O-glycosylated peptides with detection in the orbitrap at a 15,000 resolution and a 300–1,000 *m/z* and 100–1,000 *m/z* scan range for each, respectively. The targeted list included *m/z* = 204.0867|z = 0, *m/z* = 138.0545 |z = 0), *m/z* = 366.1396|z = 1), *m/z* = 163.0601|z = 1, *m/z* = 274.0921|z = 1, and *m/z* = 292.1026|z = 1.

Data were processed using Proteome Discoverer v2.4 (Thermo Scientific) with a SEQUEST HT search against the UniProtKB/Swiss-Prot Human Proteome (02/2021) and common contaminants (theGPM.org) using a 5 ppm precursor mass tolerance and a 0.02 Da fragment tolerance. Dynamic modifications included in the search were limited to oxidation [M], deamidation [NQ], and acetylation, while carbamidomethylation was the only static modification utilized for an initial search and an expanded PTM space including the above plus HexNAc. Peptides and proteins were filtered at a 1% FDR using a target-decoy approach with a minimum of two peptides per protein. Relative protein quantitation was based on an average of the top three unique peptides assigned to each protein.

### ETD MS identification of O-GlcNAc sites on A4

ETD MS was carried out at the University of Georgia Complex Carbohydrate Research Center. The target gel band containing protein was excised from the wet polyacrylamide gel and then de-stained using acetonitrile and 50 mM ammonium bicarbonate. Samples were further reduced and alkylated with DTT (25 mM, 30 min at 50°C) and iodoacetamide (90 mM, 20 min at room temperature in dark), respectively. Reduced and alkylated proteins were digested with trypsin, chymotrypsin, and elastase individually or in combination with trypsin and α-lytic protease (NEB) (1:20 ratio) at 37°C for 16 h. The peptides from digested proteins were extracted and subjected to LC-MS/MS analysis (60 min) on an Orbitrap Eclipse mass spectrometer (ThermoFisher), as previously described (54). For site identification, HCD-induced ETD was performed. The acquired LC-MS/MS data were processed with Byonic software (v4.0.12) and searched against the provided protein sequence with six most common O-glycans and Cys (S-GlcNAc) modification, as described (55). The precursor mass tolerance was set to 10 ppm, and fragment mass tolerances were set to 15 ppm and 20 ppm for HCD and ETD, respectively. Additional modifications including deamidation of N and Q, carboxymethylation of C, and oxidation of M were also included in the search. All reagents were purchased from Sigma unless otherwise mentioned. Mass spectrometric data acquisition was performed on a Thermo Scientific Orbitrap Eclipse Tribrid mass spectrometer attached to a Nano-LC system.

### OGA and OGT inhibitors

Thiamet G was purchased from Sigma and dissolved in H_2_O. OSMI-4 was purchased from MedChemExpress and dissolved in DMSO. The inhibitors were added into the medium at the designated concentrations.

### OGT degradation

MEF-ΔOGT-FKBP12^F36V^-OGT cells were provided by Suzanne Walker (38) and grown in DMEM supplemented with 15% FBS. OGT degradation was induced by 500 nM dTAG-13 (Bio-techne) during a 2 h preincubation and maintained during the entire infection.

### VACV entry assay

The recombinant VACV WRvFire, which expresses firefly luciferase under the control of a synthetic early/late VACV promoter, was previously described, and we adapted this protocol to assess VACV entry in the context of A4 O-GlcNAcylation deficiency (56, 57). WRvFire was purified from MEF-iOGT cells treated with or without dTAG by performing two rounds of sedimentation through a 36% sucrose cushion, yielding either wild-type WRvFire or A4 O-GlcNAcylation-deficient WRvFire. Viral titers were determined by plaque assay on BS-C-1 cells. For the entry assay, HeLa cells were seeded in 12-well plates and chilled to 4°C prior to virus adsorption. Purified WRvFire MVs were adsorbed in cold EMEM supplemented with 2.5% FBS for 1 hour at 4°C. Following adsorption, cells were washed with cold PBS to remove unbound virions and then incubated in pre-warmed EMEM containing 2.5% FBS at 37°C for either 1 or 2 h to allow viral entry. After incubation, cells were washed with PBS and lysed using Cell Culture Lysis Reagent (Promega) for 30 min at room temperature with gentle agitation. Luciferase activity in the lysates was measured using a Berthold Sirius luminometer (Berthold Detection Systems) according to the manufacturer’s instructions (Promega).

### Reproducibility and statistical analysis

All experimental observations, including virus yields and plaque sizes, were confirmed by fully independent experiments conducted in triplicate. Data are presented as means, with standard deviations depicted as error bars. Statistical analyses were performed using GraphPad Prism (version 10). For comparative assessments, an ANOVA multiple comparison test was employed.

## Data Availability

Materials and data that are reasonably requested will be made available in a timely fashion, at reasonable cost, and in limited quantities to members of the scientific community for noncommercial purposes. The mass spectrometry proteomics data have been deposited to the ProteomeXchange Consortium via the PRIDE partner repository (http://www.ebi.ac.uk/pride) with the data set identifier PXD062753 (10.6019/PXD062753).
